# Increasing Type 1 Poliovirus Capsid Stability by Thermal Selection

**DOI:** 10.1128/JVI.01586-16

**Published:** 2017-01-31

**Authors:** Oluwapelumi O. Adeyemi, Clare Nicol, Nicola J. Stonehouse, David J. Rowlands

**Affiliations:** School of Molecular and Cellular Biology, Faculty of Biological Sciences and Astbury Centre for Structural Molecular Biology, University of Leeds, Leeds, United Kingdom; University of Pittsburgh School of Medicine

**Keywords:** VLP, heat stable, poliovirus

## Abstract

Poliomyelitis is a highly infectious disease caused by poliovirus (PV). It can result in paralysis and may be fatal. Integrated global immunization programs using live-attenuated oral (OPV) and/or inactivated (IPV) PV vaccines have systematically reduced its spread and paved the way for eradication. Immunization will continue posteradication to ensure against reintroduction of the disease, but there are biosafety concerns for both OPV and IPV. They could be addressed by the production and use of virus-free virus-like particle (VLP) vaccines that mimic the “empty” capsids (ECs) normally produced in viral infection. Although ECs are antigenically indistinguishable from mature virus particles, they are less stable and readily convert into an alternative conformation unsuitable for vaccine purposes. Stabilized ECs, expressed recombinantly as VLPs, could be ideal candidate vaccines for a polio-free world. However, although genome-free PV ECs have been expressed as VLPs in a variety of systems, their inherent antigenic instability has proved a barrier to further development. In this study, we selected thermally stable ECs of type 1 PV (PV-1). The ECs are antigenically stable at temperatures above the conversion temperature of wild-type (wt) virions. We have identified mutations on the capsid surface and in internal networks that are responsible for EC stability. With reference to the capsid structure, we speculate on the roles of these residues in capsid stability and postulate that such stabilized VLPs could be used as novel vaccines.

**IMPORTANCE** Poliomyelitis is a highly infectious disease caused by PV and is on the verge of eradication. There are biosafety concerns about reintroduction of the disease from current vaccines that require live virus for production. Recombinantly expressed virus-like particles (VLPs) could address these inherent problems. However, the genome-free capsids (ECs) of wt PV are unstable and readily change antigenicity to a form not suitable as a vaccine. Here, we demonstrate that the ECs of type 1 PV can be stabilized by selecting heat-resistant viruses. Our data show that some capsid mutations stabilize the ECs and could be applied as candidates to synthesize stable VLPs as future genome-free poliovirus vaccines.

## INTRODUCTION

Poliomyelitis (polio) is an incapacitating human disease caused by three structurally similar but antigenically distinct serotypes of poliovirus (PV) termed PV-1, PV-2, and PV-3 (1–3). Live-attenuated oral PV vaccines (OPV) and inactivated-PV vaccines (IPV) have been used in massive integrated global immunization schemes, culminating in a feasible strategic-endgame plan for polio eradication (4). This has not occurred without some major setbacks. OPV has been known to regain virulence through reversion or recombination (5), resulting in individual cases of vaccine-associated paralytic poliomyelitis (VAPP) or polio outbreaks due to circulating vaccine-derived PV (cVDPV). Chronic carriers of cVDPVs may serve as virus depots and reseed populations. Although the use of IPV avoids these issues, its production involves the growth of large amounts of virus with the attendant risk of accidental release in a polio-free world (6, 7). In addition, although IPV effectively protects recipients from disease, it fails to prevent replication and subsequent transmission of virus in a population (8, 9).

**FIG 1:**
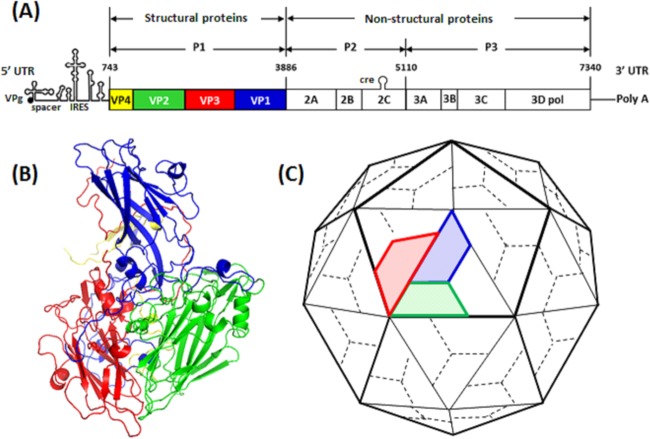
Poliovirus genome organization and morphogenesis. (A) Genomic structure of PV-1 showing structural protein genes in blue (VP1), green (VP2), red (VP3), and yellow (VP4). (B) Ribbon model of a PV protomer showing relative positions of the capsid proteins. (C) Cartoon model of the PV capsid, with a pentamer outlined. Five protomers assemble into a pentamer, 60 of which form a procapsid. Virion maturation involves cleavage of VP0 into VP2 and VP4. The diagrams are not to scale.

PV has a 7.5-kb positive-sense RNA genome enclosed in a 30-nm icosahedral capsid that comprises 60 copies each of viral proteins VP1, VP2, VP3, and VP4 (10, 11). The capsid is reinforced by a network of internal N termini of VP1, VP2, and VP3, together with the internal protein VP4 (1) (Fig. 1). All three PV serotypes have been shown to possess four neutralizing antigenic sites, termed N-AgI (VP1 residue positions 90 to 104), N-AgII (VP1 residues 220 to 222 and VP2 residues 164 to 175), and N-AgIIIA (VP1 residues 286 to 290) and N-AgIIIB (VP3 residues 58 to 60 and 71 to 79) (12–14). In PV-1, N-AgII and N-AgIII have been shown to have immunodominance over N-AgI (12, 13).

Structural proteins VP1, VP2, and VP3 differ in amino acid sequence but form structurally similar wedge-shaped, 8-stranded β-barrels, comprising two antiparallel β-sheets essential for virion stability (1, 15). Within the 8-stranded β-fold of VP1, there is a highly conserved pocket that contains a lipid moiety (pocket factor). This feature is intimately involved in the conformational changes associated with uncoating (3). The crystal structure of the PV capsid shows star-shaped mesas at the 5-fold axes and three-bladed propeller-like structures at the 3-fold axes. Surrounding each star-shaped mesa is a canyon (1), which is the site of attachment to the cellular PV receptor, CD155 (16, 17).

In the course of infection, attachment of a 160S PV particle to the CD155 receptor triggers conformational changes in the virion capsid that result in formation of 135S particles (18) and release of the pocket factor (1). The virion enters the cell through cell-mediated endocytosis, after which the viral genome is released into the host cytoplasm (19). The viral genome is translated into a polyprotein that is cleaved by virus-encoded proteases to form three precursors, P1, P2, and P3; further proteolytic cleavage of P1 results in three structural proteins, VP1, VP3, and VP0 (a precursor of VP2 and VP4). In the early stages of PV morphogenesis, VP1, VP3, and VP0 assemble into a protomer (20–22); 5 protomers assemble to form a pentamer, and 12 pentamers can assemble into an 80S procapsid. In the presence of viral RNA, cleavage of VP0 results in mature VP2 and VP4 (20).

Empty PV capsids (ECs) with intact VP0 have been isolated from infected cells (1, 20). These ECs have antigenic features that are similar/identical to those of mature virions, and although their role in the viral life cycle has been debated and remains unclear, a study carried out on enterovirus 71 (EV71) ECs suggests that they may serve as decoys to neutralizing antibodies (23). Addition of guanidine hydrochloride (GuHCl) to PV-infected cells inhibits RNA synthesis and leads to accumulation of ECs (18, 24). These genome-free PV ECs could serve as potential virus-like particle (VLP) vaccines. Attempts have been made to produce PV VLPs in various recombinant expression systems (e.g., insect, Saccharomyces cerevisiae, or human cells) (25–27); however, wild-type (wt) PV empty capsids are very unstable and readily switch antigenicity from a native form (termed N or D) to a nonnative or heated form (termed H or C) (28). Although pocket-binding compounds have been shown to stabilize the PV VLPs (27, 29), their use is incompatible with commercial vaccine production (30, 31).

An alternative strategy could involve the generation of stabilized capsids that undergo antigenic conversion less readily, e.g., by introduction of mutations. RNA viruses are prone to continual mutagenesis due to the nonproofreading RNA-dependent RNA polymerase (RdRp), which allows rapid evolution under selection pressure (32, 33). This has enabled selection of heat-resistant PVs (34, 35). In this study, we attempted to generate stabilized ECs of PV-1 by first selecting for thermally stable PV-1 mutants. The potential of ECs produced by these mutant viruses for the development of a PV VLP vaccine is discussed.

## RESULTS

### Selection of thermally stable PV-1 mutants.

**FIG 2:**
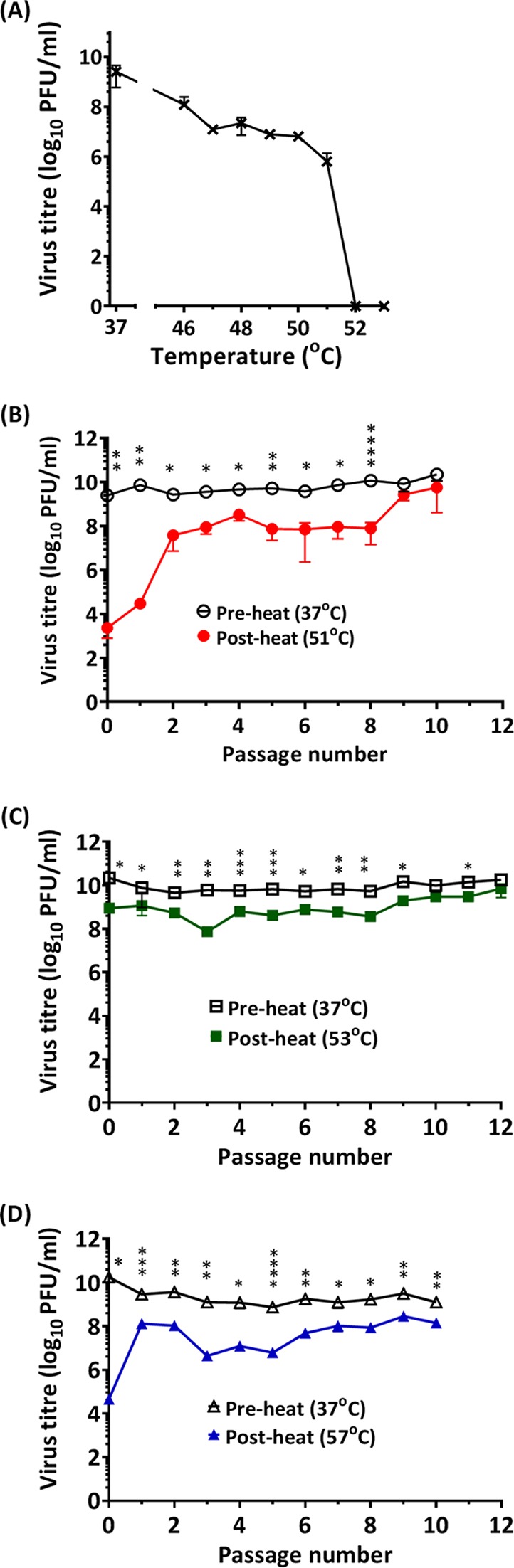
Evolution of heat-resistant PV through repeat cycles of selection and passage. (A) Wt PV-1 was incubated at a range of temperatures for 30 min and cooled to 4°C. The surviving pool of viruses was evaluated by plaque assay on HeLa cells. (B) Wt PV-1 was incubated at 51°C for 30 min, which resulted in 99.99% loss of infectivity, and immediately cooled to 4°C. The surviving pool of viruses was passaged at 37°C. After each cycle of passage, virus titers (pre- and postheating) were determined by plaque assays. The selection cycles were repeated until the pre- and postheating virus titers were approximately equal (*n* = 3 ± standard deviation [SD]; *, *P* < 0.05; **, *P* < 0.001; ****, *P* < 0.00001). (C) After 10 cycles of thermal selection at 51°C and passage at 37°C, thermal pressure was increased to 53°C with 12 successive passages at 37°C. The pre- and postheating titers were statistically different from passage 0 until passages 9 and 11 (*n* = 3 ± SD; *, *P* < 0.05; **, *P* < 0.001; ***, *P* < 0.0001). (D) After selection at 53°C, thermal selection pressure was subsequently increased to 57°C with 10 successive passages. The pre- and postheating titers were statistically different from passage 0 until passage 10 (*n* = 3 ± SD; *, *P* < 0.05; **, *P* < 0.001; ***, *P* < 0.0001; ****, *P* < 0.00001). Three titrations of the same selected pool were analyzed at each temperature.

The construction of a genome-free PV VLP vaccine has been hindered in part by the instability of wt PV-1 genome-free ECs. Therefore, we first sought to select PV mutants with increased thermal stability using repeated thermal inactivation followed by passage of surviving virus, focusing on PV-1. At 51°C, the infectivity of wt PV-1 was reduced by 99.99% compared to the unheated control, and it was chosen for the initial selection temperature (Fig. 2A). Heat-resistant PV-1 was selected by sequential heating to 51°C, followed by passage at 37°C (Fig. 2B). To assess the genetic stability of thermally selected PV-1, virus was titrated after each passage in the presence or absence of selection pressure. Thermal selection was repeated for 10 successive passages until there was no statistically significant difference between the postheating and preheating viral titers. This thermally selected PV-1 pool was termed VS51. Subsequently, the VS51 virus pool was used for 12 sequential selections at 53°C, followed by passages at 37°C with pre- and postheating virus titers determined after each passage, as before (Fig. 2C). Following 12 successive cycles of heating and passage, there was no statistically significant difference between the postheating and preheating virus titers. This new thermally selected pool was termed VS53. Finally, the VS53 pool was utilized for selection at 57°C, as before (Fig. 2D). After 10 successive passages, the final virus pool was termed VS57. In contrast to selection at 51°C and 53°C, there was a statistically significant fitness cost of 1-log_10_-unit reduction in infective titers even after further selection for two rounds (data not shown), due to selection pressure at 57°C. These 3 pools of thermally selected viruses, VS51, VS53, and VS57, were further characterized.

### Thermal resistance of heat-selected viruses.

**FIG 3:**
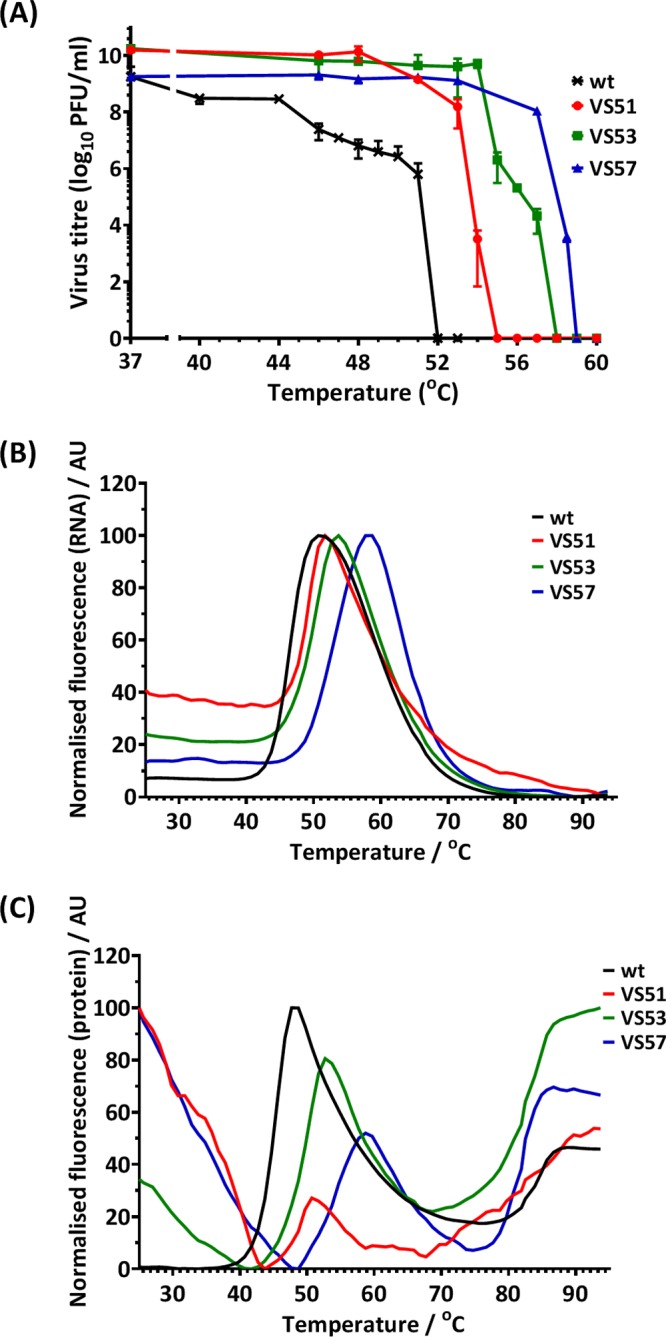
Thermal-resistance profile of heat-selected virus pools. (A) Pools of PV-1 selected at 51°C (VS51), 53°C (VS53), and 57°C (VS57) were incubated at a range of temperatures between 37°C and 60°C for 30 min and immediately cooled to 4°C. Titers were determined by plaque assays on HeLa cells. The data represent titers at each temperature (*n* = 3 ± SD; *P* < 0.0001). Wt PV-1 and thermally selected purified virus samples were examined by differential scanning fluorometric assays (PaSTRy) using SYTO9 nucleic acid-binding dye and SYPRO orange protein-binding dye as described by Walter et al. (36). (B) Relative fluorescence of SYTO9 (*n* = 3 ± SD; *P* < 0.0001). (C) Relative fluorescence of SYPRO orange (*n* = 3 ± SD; *P* < 0.001). The error bars in panels B and C were omitted for clarity. AU, arbitrary units.

The ability of VS51, VS53, and VS57 to withstand elevated temperatures was assessed using two methods: thermal inactivation (i.e., biological assay to measure virus infectivity by plaque assays) and particle stability thermal-release assay (PaSTRy) (i.e., a biochemical assay to measure capsid stability). Thermal-inactivation assays showed that the selected viruses (VS51, VS53, and VS57) maintained infectivity at higher temperatures than the wt. Complete thermal inactivation of the wt occurred at 52°C, while the selected viruses were inactivated at higher temperatures (Fig. 3A). Data for the selected viruses (VS51, VS53 and VS57) were significantly different (*P* < 0.0001) from those for the wt.

PaSTRy can directly determine the thermal stability of virus particles without the need for virus growth (36). The assay employs two fluorescent dyes: SYTO9, which binds nucleic acid and indicates accessibility to the viral RNA, and SYPRO orange, which binds hydrophobic amino acid residues and indicates unfolding/denaturation of the capsid proteins. Maximal fluorescence of SYTO9 occurred at 51°C (wt), 52°C (VS51), 54°C (VS53), and 58°C (VS57) (Fig. 3B). The results for the selected viruses (VS51, VS53, and VS57) were significantly different (*P* < 0.0001) from those for the wt.

SYPRO orange had two peaks. One indicated conformational change associated with capsid expansion and exposure of hydrophobic residues at 48°C (wt), 51°C (VS51), 53°C (VS53), and 59°C (VS57). The second peak, at around 85°C in all the virus samples, has been previously associated with denaturation (36). The data for the selected viruses (VS51, VS53, and VS57) were significantly different from those for the wt at the 0.001 significance level. Both SYTO9 and SYPRO orange PaSTRys confirm the observations from infectious thermal-inactivation assays and demonstrate that thermally selective virus pools could withstand elevated temperatures compared to wt PV-1.

The results suggest that thermal stability may be associated with a synchronized temperature for RNA release (*T_R_*) and the first protein melting event (*T_m_*), which may coincide with thermal inactivation. The data further show that for all the selected viruses, an increase in *T_R_* was matched by an increase in *T_m_*. In contrast, hepatitis A virus (HAV), a more thermally stable picornavirus, was shown to have a *T_R_* higher than its *T_m_* by PaSTRy (37). This suggests that as PV attains higher thermal stability, RNA release and capsid alteration may occur in synchrony.

### Purification and analysis of ECs.

**FIG 4:**
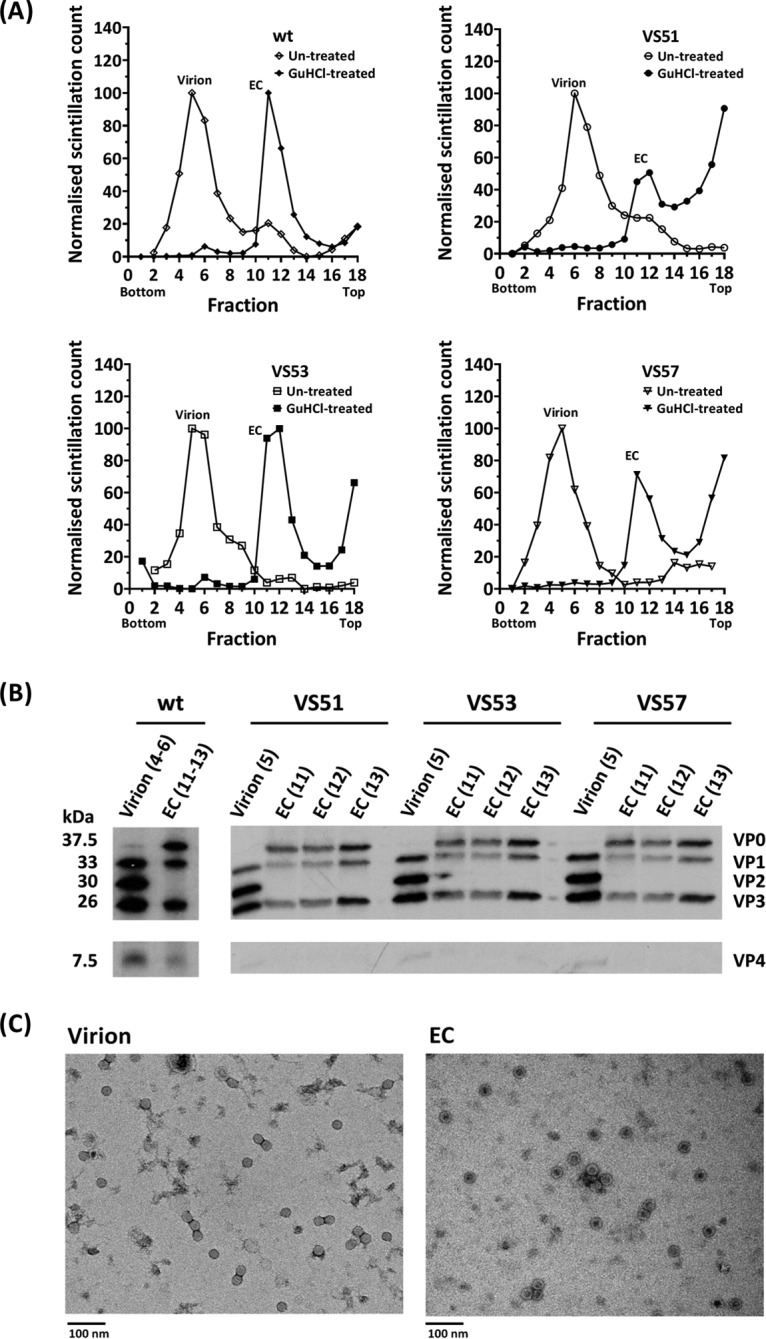
Generation and purification of virions and ECs. HeLa cells were infected with the wt, VS51, VS53, and VS57. Virus capsid proteins were radiolabeled with [^35^S]Cys/Met at 2.75 h postinfection and incubated at 37°C. For EC preparation, 2 mM GuHCl was added at 3.15 h postinfection. The harvested particles were purified by ultracentrifugation. (A) Scintillation counts of fractions of 15 to 45% sucrose density gradients of the wt, VS51, VS53, and VS57. The gradients were fractionated into 300-μl aliquots, and 3% of each fraction was counted by scintillation. (B) Autoradiograph of proteins from virion and EC sucrose density gradient peak fractions of the wt, VS51, VS53, and VS57. Samples from virion and EC peaks were boiled in SDS loading buffer. Proteins were separated by SDS-PAGE, and [^35^S]Cys/Met-radiolabeled virus bands were detected by autoradiography. (C) Electron micrographs of virion and EC peaks of the wt. Virion and EC peaks were harvested and dialyzed into 10 mM HEPES, 100 mM NaCl, 1 mM EDTA, 1 mM DTT, pH 6.4, and visualized by negative-staining transmission electron microscopy.

Low concentrations of GuHCl have been shown to inhibit PV RNA synthesis (29), resulting in accumulation of ECs (18, 24). To isolate ECs from the thermostable viruses, 2 mM GuHCl and [^35^S]Cys/Met were added to infected cell cultures at the times of peak protein and RNA synthesis, respectively. Subsequently, particles were harvested from lysed cells and purified through sucrose density gradients. Virion and EC peaks were observed (Fig. 4A). As expected, a prominent virion peak and a smaller EC peak were detected in the untreated-sample gradient, while a predominant EC peak was detected in the GuHCl-treated-sample gradient. Transmission electron micrographs of dialyzed gradient peak fractions (Fig. 4C) confirmed that GuHCl treatment resulted in accumulation of ECs that were permeable to the stain, which can readily penetrate (38) a “breathing” capsid (39), whereas stain did not penetrate the isolated virions due to exclusion by the RNA densely packed within the capsid (38). Analysis of the gradient peak fractions by autoradiography following SDS-PAGE (Fig. 4B) confirmed that virions contained the mature cleavage products VP2 and VP4, whereas VP0 remained uncleaved in the EC peak fractions, as expected.

### Antigenic stability of thermally selected particles.

**FIG 5:**
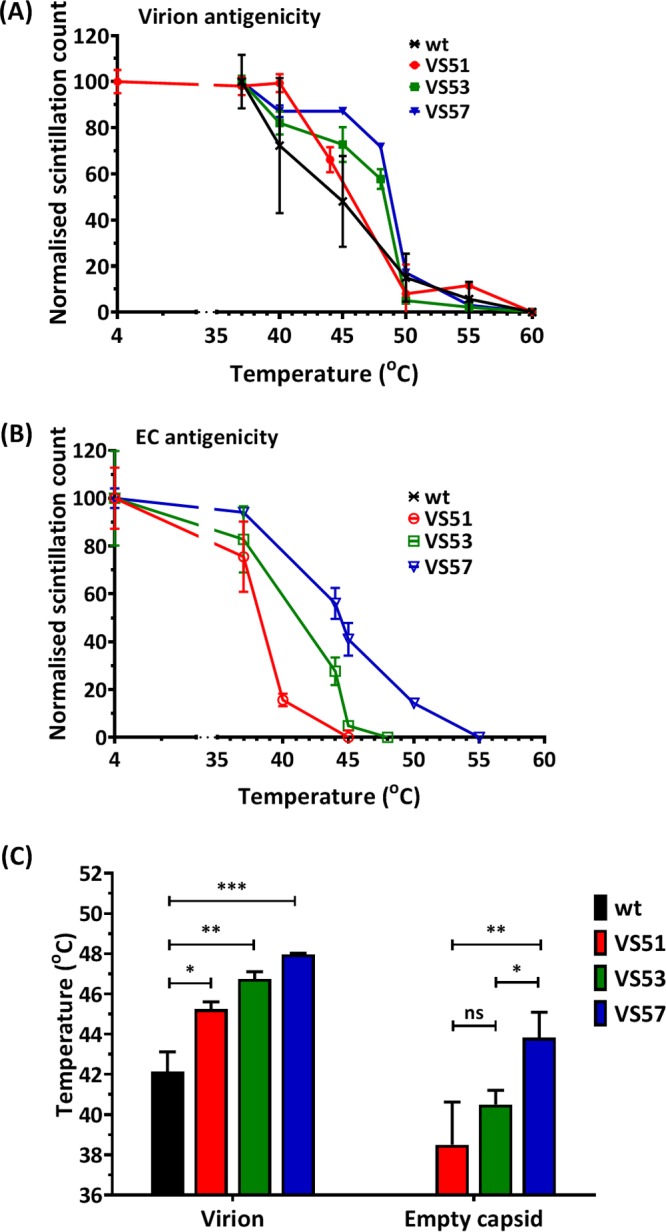
Antigenic-stability profile of heat-selected virus pools. Pools of thermally selected PV-1 at 51°C (VS51), 53°C (VS53), and 57°C (VS57) were radiolabeled with [^35^S]Cys/Met and purified by differential and sucrose gradient centrifugation. Virion and EC peaks were identified by scintillation counting. Virion and EC fractions were harvested and incubated at a range of temperatures for 30 min and then cooled to 4°C. Heat-treated N-specific particles were immunoprecipitated using MAb 234. (A) Normalized scintillation counts of heat-treated virions (*n* = 3 ± SD; *P* < 0.05). (B) Normalized scintillation counts of heat-treated EC particles (*n* = 3 ± SD). It was not possible to compare values to the wt, as they convert completely to the H/C form at 10°C. (C) The temperatures at which 50% of the N/D antigenicity was converted to the H/C form were estimated for virion particles of selected viruses (VS51, VS53, and VS57) compared to the wt (*, *P* < 0.05; **, *P* < 0.001; ***, *P* < 0.0001; ns, not significant).

When heated above 50°C, wt PV-1 undergoes an antigenic switch from a native (termed N or D) to a nonnative or heated (termed H or C) form due to conformational changes in capsid proteins (35, 40). ECs have been shown to undergo similar conformational antigenic changes at 10°C (28). We anticipated that the thermally selected viruses would undergo the antigenic switch at elevated temperatures. To investigate this, the antigenic properties of [^35^S]Cys/Met-labeled virions or ECs of wt PV-1, VS51, VS53, and VS57 were assessed by immunoprecipitation with antibodies that distinguished between the two antigenic forms. Two monoclonal antibodies (MAbs) were employed for immunoprecipitation, MAb 234, which recognizes native (N/D) virions, and MAb 1588, which recognizes nonnative (H/C) particles (2, 14, 41). The temperature at which 50% of the N/D antigenicity was converted to the H/C form was estimated by scintillation counting of the relative amounts of [^35^S]Cys/Met-radiolabeled particles precipitated by the different antibodies (Fig. 5). Wt PV-1 virions lost 50% of the N/D antigenicity at 42°C and showed a complete switch from N/D to H/C antigenicity at 50°C, as previously reported (35, 40, 42). In contrast, VS51, VS53, and VS57 lost 50% of the N/D antigenicity at 45°C, 47°C, and 48°C, respectively (Fig. 5). Antigenicity curves of the viruses (VS51, VS53 and VS57) were statistically significantly different from that of the wt at the 0.05 significance level.

Wt PV-1 ECs do not retain native antigenicity above 10°C (28), and therefore, such samples were not analyzed here. The thermally selected viruses were shown to have N/D antigenic ECs, with the switch to H/C antigenicity occurring at about 38°C (VS51), 40°C (VS53), or 44°C (VS57). VS57 was significantly more stable than VS53 (*P* < 0.05) and VS51 (*P* < 0.001); however, VS51 and VS53 were not significantly different (Fig. 5).

Together, these data demonstrate that both full virions and ECs of the thermally selected viruses had an increased native antigenic tolerance for elevated temperatures.

### Identification of mutations present in heat-selected viral capsids.

**FIG 6:**
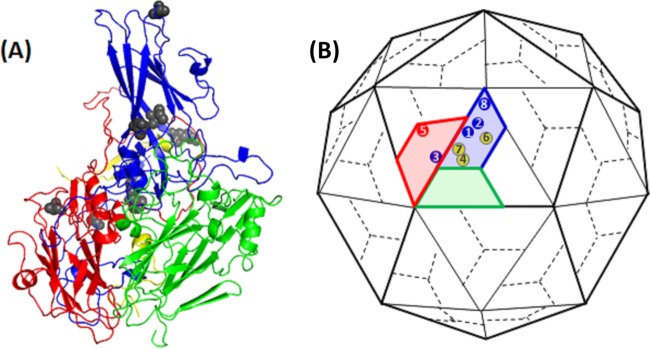
Positions of common mutations. (A) Ribbon model of a PV protomer. (B) Cartoon model of PV capsid, with VP1 (blue), VP2 (green), VP3 (red), and VP4 (yellow). The positions of VP1 mutations (1, V1087A; 2, I1194V; 3, A1026T; and 8, S1097P), a VP3 mutation (5, C3175A), and VP4 mutations (4, F4046L; 6, R4034S; and 7, D4045V) are indicated. Most mutations occurred at residues buried within the capsid. The diagrams are not drawn to scale.

To identify the capsid mutations in thermally selected virus pools, genomic RNA was extracted, the P1 coding region was amplified by reverse transcription-PCR, and purified amplicons were sequenced. Additionally, amplicons were cloned into a plasmid, individual colonies were picked, and capsid-encoding (P1) sequences were determined. The nucleotide sequences of PCR amplicons and individual clones (*n* = 3) corresponded. Nucleotide sequence alignment of the thermally selected viruses, VS51, VS53, and VS57, against wt PV-1 (Mahoney) (NCBI GenBank reference sequence V01149.1) (43) showed various nonsynonymous capsid mutations (Table 1). The positions of capsid mutations of VS51, VS53, and VS57 were mapped onto the structural model of PV-1 (Mahoney) (Protein Data Bank [PDB] ID, 1ASJ) (22) using PyMOL (44) (Table 2 and Fig. 6). PV-1 (Mahoney), PV-2 (Sabin), and PV-3 (Leon) were aligned (45) and compared to identified mutations. The release of the PV pocket factor is an important capsid-destabilizing step that precedes uncoating the viral genome (1). The PV pocket is a highly conserved region among the three PV serotypes and includes residues 1087 and 1194 (3, 45). These mutations (V1087A and I1194V) were identified in all three thermally selected virus pools. A mutation identified in VS57 (S1097P) changes to the residue found in wt PV-2 and PV-3. This is an external residue on the 5-fold pentameric vertex. All the other mutations were located on intersubunit interfaces within the viral capsid.

**TABLE 1:** Selected capsid mutations

Selected virus/SDM construct	Capsid mutation(s)/amino acid substitution(s)
VS51	V1087A, I1194V
VS53	A1026T, V1087A, I1194V, F4046L
VS57	V1087A, S1097P, I1194V, C3175A, R4034S, D4045V
I1194V	I1194V
PV51δ	V1087A
PV51	V1087A, I1194V
PV53δ	A1026T, V1087A, F4046L
PV53	A1026T, V1087A, I1194V, F4046L
PV57δ	V1087A, S1097P, C3175A, R4034S, D4045V
PV57	V1087A, S1097P, I1194V, C3175A, R4034S, D4045V

**TABLE 2:** Positions of mutations within virus capsids

Mutation	Residue position	Residue^*a*^	
		PV-2 (Sabin)	Pv-3 (Leon)
A1026T	VP1; internal surface of the virus capsid; close to the N-terminus of VP1	S	S
V1087A	VP1; B strand of the VP1 β-barrel; outer surface of the virus pocket	*	*
S1097P	VP1; capsid surface at the 5-fold interpentameric vertex; residue resides on B-C loop of the VP1 β-barrel.	P	P
I1194V	VP1; within the virus pocket; G strand of the VP1 β-barrel	*	*
C3175A	VP3; surface G-H loop of the VP3 β-barrel; a capsid surface residue, located on the surface G-H loop of the VP3 β-barrel; situated between monomers within a pentamer	*	*
R4034S	VP4; internally located in virus capsid	*	K
D4045V	VP4; internally located in virus capsid	*	*
F4046L	VP4; internally located in virus capsid	*	Y

a *, residue homology between wt PV-1 and the respective serotype (i.e., PV-2 and/or PV-3).

### Characterization of common pocket mutations, V1087A and I1194V.

**FIG 7:**
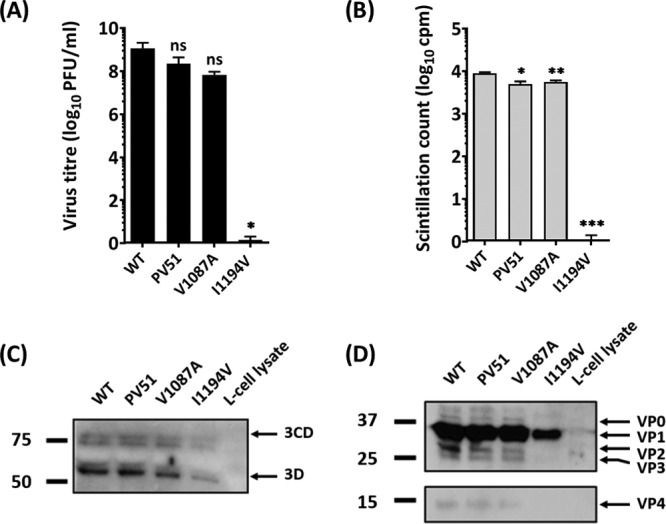
Characterization of pocket mutations V1087A and I1194V. Two common mutations that occurred in all mutants were reintroduced by site-directed mutagenesis into an infectious clone of wt PV-1 individually and in combination (PV51). Viral RNA was generated by T7 transcription. A total of 5 × 10^7^ L cells were transfected with 5 μg RNA and incubated at 37°C. Virus proteins were radiolabeled with [^35^S]Cys/Met and purified by differential and sucrose gradient centrifugation. (A) Virus titers were determined by plaque assays on HeLa cells (*n* = 3 + SD; *, *P* < 0.05). (B) Virions were immunoprecipitated with N/D-specific (MAb 234) and H/C-specific (MAb 1588) monoclonal antibodies and counted by scintillation. The sum of the radioactivity in immunoprecipitated particles from 30% of virus peak fractions is presented and compared to that of the wt (*n* = 3 + SD; *, *P* < 0.05; **, *P* < 0.001; ***, *P* < 0.0001). (C and D) Transfected cell lysates were blotted against anti-3CD (C) and polyclonal P1 antibody (SH-16) (D). The numbers on the left are in kilodaltons.

The VP1 pocket mutation V1087A was reported previously to increase the thermal stability of PV-1 and PV-2 (35). In order to characterize the two pocket mutations (V1087A and I1194V) that occurred in all the selected virus pools in this study (VS51, VS53, and VS57), they were introduced individually or together into an infectious clone of wt PV-1. Viral mRNA was generated from infectious clones and transfected into mouse L cells, and capsid proteins were radiolabeled as before. Infectious virions were recovered from the wt, V1087A, and PV51, which is an infectious clone of PV-1 that includes the mutations selected at 51°C (i.e., V1087A and I1194V in combination). However, I1194V produced no infectious particles (Fig. 7A). Particles were recovered from cell supernatants and lysates and purified as before. Virion peak fractions were immunoprecipitated using N/D-specific MAb 234 or H/C-specific MAb 1588 and analyzed by scintillation counting (Fig. 7B) and Western blotting (Fig. 7C and D). All RNA transcripts produced capsid proteins and the replication proteins, 3D and 3CD, but I1194V produced much lower levels (Fig. 7C and D) for reasons that are currently unclear. However, it should be noted that a loading control was not included. N/D antigenic and H/C antigenic particles were immunoprecipitated following transfection with RNA transcripts of the wt, VS51, and V1087A, but no immunoprecipitated particles resulted from I1194V (Fig. 7B).

### Effects of I1194V mutation on selected mutants.

**FIG 8:**
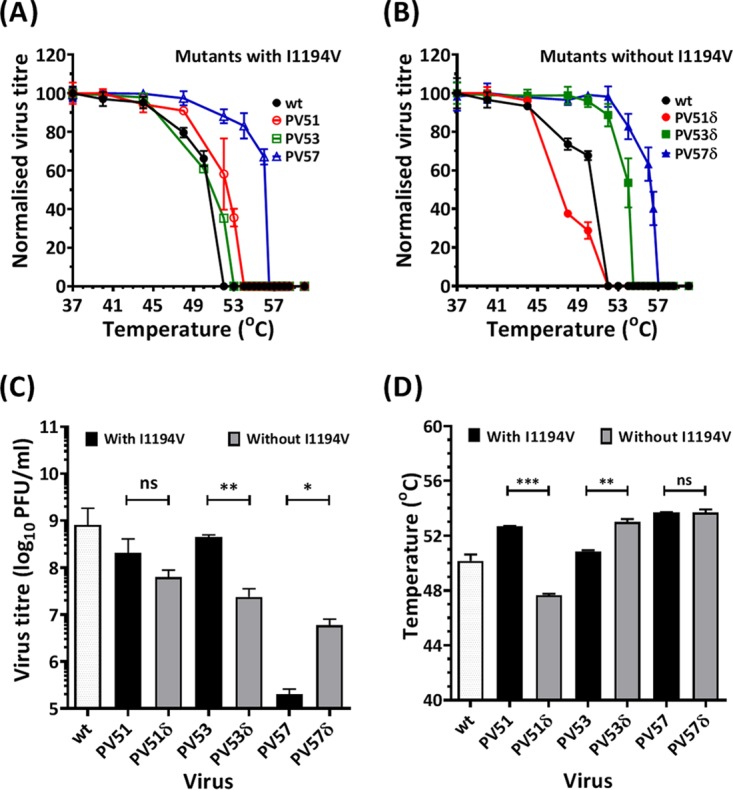
Infectivity of SDM-constructed mutants. Mutant viruses were constructed individually and in combinations by SDM, with and without I1194V. PV51δ (V1087A), PV53δ (A1026T, V1087A, F4046L), and PV57δ (V1087A, S1097P, C3175A, R4034S, D4045V) were generated and compared with constructs in which I1194V was present, i.e., PV51 (V1087A, I1194V), PV53 (A1026T, V1087A, F4046L, I1194V), and PV57 (V1087A, S1097P, C3175A, R4034S, D4045V, I1194V). A total of 1 × 10^7^ mouse L cells were transfected with 5 μg of *in vitro*-transcribed RNA. Posttransfection, the supernatant was harvested and the cells were freeze-thawed. Samples were heat treated at a range of temperatures, and virus titers were determined by plaque assays using HeLa cells. (A) Normalized thermal-inactivation assays for SDM mutants with I1194V (*n* = 3 ± SD; *P* < 0.00001). (B) Normalized thermal-inactivation assays for SDM mutants without I1194V (*n* = 3 ± SD; *P* < 0.00001). (C) Input virus titers (*n* = 3 ± SD; *, *P* < 0.05; **, *P* < 0.001). (D) 50% inhibitory concentration [IC_50_] temperatures for thermal-inactivation curves of mutants with and without I1194V (*n* = 3 ± SD; **, *P* < 0.001; ***, *P* < 0.0001).

Given the effect of I1194V in isolation on virion production, we sought to further investigate the effects of the mutation. Site-directed mutagenesis (SDM) was used to generate clones PV51, PV53, and PV57, both with and without (δ) the mutation I1194V (Table 1), and the corresponding RNA transcripts were used to transfect L cells. The infectivity titers of the resulting virus harvests were determined by plaque assays on HeLa cells (Fig. 8). Viruses recovered following transfection of RNA from the SDM-derived infectious clones were shown to lose infectivity at 52°C (wt), 54°C (PV51), 53°C (PV53), and 56°C (PV57). It was observed that PV51 was more thermally stable than its corresponding delta (without I1194V) mutant (*P* < 0.0001). PV51 was significantly more stable than the wt (*P* < 0.05) and PV53 (*P* < 0.001); however, PV53 was not significantly different from the wt. PV57 was significantly (*P* < 0.05) more stable than all of the viruses tested (Fig. 8A). PV53δ could significantly (*P* < 0.0001) withstand higher temperatures than the corresponding nondelta mutant (PV53), while PV57 and its corresponding delta mutant had similar inactivation temperatures (Fig. 8A and B). Removal of I1194V resulted in a 4-fold but nonsignificant decrease in the titer of PV51 and a 13-fold, significant (*P* < 0.001), decrease of PV53, respectively. Removal of I1194V from VS57, however, resulted in a 40-fold, significant (*P* < 0.05), increase in the virus titer (Fig. 8C). Although PV51 was more thermally stable than PV53, removal of I1194V from PV53 (to create PV53δ) resulted in a more thermally stable virus with a profile similar to that of VS53 (Fig. 8D).

### Thermal-stability profiles of candidate mutant viruses.

**FIG 9:**
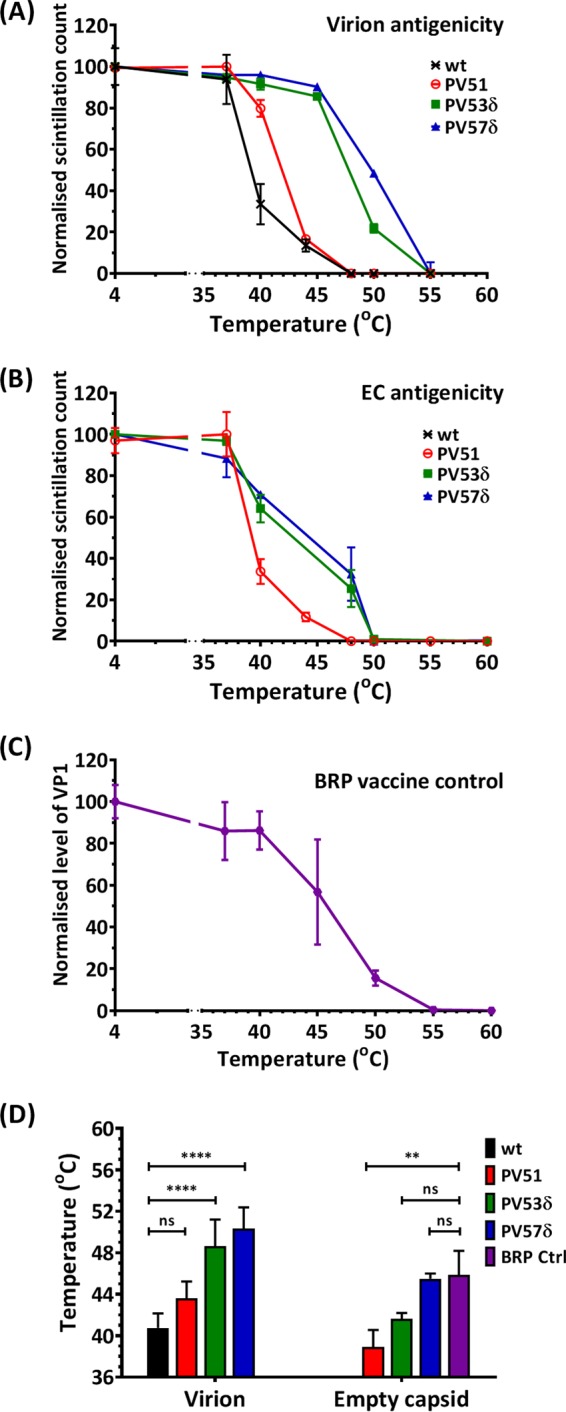
Thermal-stability profiles of virus mutants. Mutations identified by thermal selection were introduced by SDM into an infectious clone of wt PV-1, individually and in combinations with and without the I1194V mutation. The wt was included for comparison. Transcribed RNAs were transfected into L cells to recover infectious virus, and ECs were accumulated by GuHCl treatment at 3.15 h posttransfection. The harvested particles were purified through 15 to 45% sucrose density gradients. The sucrose was removed by dialysis, and virions and ECs were dialyzed out of the sucrose and incubated at a range of temperatures (from 37°C to 60°C) for 30 min and cooled to 4°C for 5 min before titration by plaque assays using HeLa cells. (A) Immunoprecipitation assay for heat-treated virions. (B) Immunoprecipitation assay for heat-treated ECs using N/D-specific (MAb 234) monoclonal antibodies. (C) Immunoprecipitation assay for heat-treated BRP vaccine controls, using N/D-specific (MAb 234) monoclonal antibodies. Samples were immunoblotted with anti-VP1 Millipore MAb 8650. Shown are normalized averages of semiquantitative densitometric plots of VP1 band intensities using ImageJ (*n* = 3 ± SD). (D) Temperatures at which 50% native (N/D) antigenicity was lost. *n* = 3 + SD. For virus, the data are compared to the wt, and for EC, to BRP (**, *P* < 0.001; ****, *P* < 0.00001).

Owing to the effects of I1194V discussed above, PV51, PV53δ, and PV57δ were taken forward for more detailed analysis. Antigenicity profiles of virion particles (Fig. 9A) showed that PV53δ and PV57δ were significantly (*P* < 0.001) more thermally stable than PV51, and both were significantly (*P* < 0.00001) more thermostable than the wt virion. The antigenic switches of virions occurred at 44°C (PV51), 49°C (PV53δ), and 50°C (PV57δ). EC antigenicity profiles (Fig. 9B) also showed that PV57δ was significantly more thermally stable than PV53δ (*P* < 0.05) and PV51 (*P* < 0.0001). For the corresponding ECs of PV51, PV53δ, and PV57δ, antigenic switches occurred at 38°C, 42°C, and 45°C, respectively (Fig. 9D). Since the wt EC is antigenically unstable, a current IPV vaccine (i.e., biological reference preparation [BRP] vaccine) was used as a control (Fig. 9C). The results showed that although PV51 was significantly (*P* < 0.001) less stable than BRP, both PV53δ and PV57δ were as stable as BRP (Fig. 9D).

## DISCUSSION

In this study, we sought to increase the thermal stability of PV-1 virions and ECs by sequential cycles of infection following thermal selection at increasing temperatures up to 57°C. We derived three virus pools with progressively increased thermostability following selection at 51°C (VS51), 53°C (VS53), and 57°C (VS57). This was confirmed by thermal-inactivation assays, which measured virus infectivity and fitness. We also measured the capsid flexibility under continuously increasing thermal stress by PaSTRy. We were able to capture the melting points of the virus capsids, which increased upon selection. We also showed that ECs generated from selected virions were antigenically more stable, withstanding temperatures up to 45°C. Through reverse genetics, we identified the mutations responsible for the increased thermal stabilities and confirmed them by reintroducing individual mutations into an infectious clone, with similar results.

The two key VP1 mutations (V1087A and I1194V) that evolved in early cycles of selection were maintained as a consistent pair in all subsequent selected candidates. In 2004, a study reported that V1087A is a heat-resistant mutation able to withstand 50°C (35), although the effect of the mutation on EC formation was not reported. Our findings suggest increased stability of V1087A in combination with I1194V. It has also been reported that mutations of VP1 residue 194 in PV-1, PV-2, and PV-3 may reduce thermal stability (46, 47). In line with this submission, our data further show that mutation of this residue to valine affects particle assembly or virus release, since the I1194V mutant was unable to produce virus. However, when I1194V was in combination with V1087A, the production of virus was restored, and capsid stability appeared to be enhanced over that with V1087A alone.

VP1 residues 87 and 194 both lie within the pocket that is known to play a role in capsid stability. These residues are located on the B and G strands of the VP1 β-barrel, respectively, and may play vital roles in EC stability. Based on the proximity of the B and G strands to antigenic binding sites on the BC loop (48), interactions between the residues may have a regulatory effect on the antigenic conformation of the capsid (2, 49). It is noteworthy that both substitutions were to smaller side chains, which may have resulted in a deeper or more spacious pocket that may assist the capsid to retain the pocket factor at higher temperatures than the wt. It has been shown that substitutions of larger residues in the pockets of EV71 and coxsackievirus A16 make the viruses more thermolabile (30). This is consistent with data reported for other enteroviruses, including PV-3 (31, 50, 51).

In the native conformation, residues 95 to 104 around the VP1 BC loop of the viral capsid contribute to antigenic sites (2, 49). Of all the mutations selected, a VP1 mutation (S1097P) occurred on a residue that lies within the PV-1 antigenic site (N-AgI) and is one of 15 residues that are well exposed on the apex loop. It is, however, interesting that the mutation to P is found in wt PV-2 and PV-3. N-AgI is the least immunodominant of all the N-Ag sites and is identifiable by H-specific MAbs even after denaturation of the capsid protein by heat treatment (12, 13).

Recently, it has been postulated that heating 160S particles results in capsid expansion that creates a greater distance between adjacent antigenic BC loops in an event that coincides with loss of native antigenicity (48, 52). In retaining the N/D antigenicity at physiological temperatures, it is tempting to suggest that both pocket mutations (V1087A and I1194V) may have triggered an internal stabilizing effect from within the capsid, thereby preventing expansion of the ECs when heated.

Further selection of VS51 generated a virus with two internal mutations, one in VP4 (F4046L) and a second in VP1 (T1026A). Further selection led to reversion of these mutations and identification of additional internal mutations, such as an interpentamer VP3 mutation (T3175A) that, in combination, stabilized the EC structure further. Owing to high rates of mutation, RNA virus populations comprise a quasispecies of genetically linked viruses distributed around a consensus sequence (53). As a population of viruses selected from VS53, the VS57 pool of viruses possibly includes viruses with T1026A and F4046L, as well as other mutations (53, 54). The existence of additional mutations in the wt pool could have been detected by deep sequencing; however, while this method provides depth of mutations, it is limited because of short reads.

Owing to the inability of the I1194V mutation to assemble particles, we assessed the effect of omitting the mutation in PV53 and PV57 mutants (i.e., PV53δ and PV57δ). Our findings showed that in the absence of I1194V, PV53δ and PV57δ had greater capsid stabilities. This may further suggest the destabilizing effects of mutating the highly conserved VP1 residue 194 on virus fitness.

It has been shown that stabilized N-specific ECs of PV are recognized by all known neutralizing monoclonal antibodies (14, 55), as well as the poliovirus receptor (18). It has been shown that PV-1 VLPs expressed in yeast are immunogenic and can elicit neutralizing antibodies comparably to N-antigenic IPV in mice (27). However, these VLPs retained the N-antigenicity only when stabilized with a pocket-binding compound to prevent antigenic switch to nonnative antigenicity. Our study shows that in the absence of capsid-stabilizing agents, the genome-free ECs can be stabilized by capsid mutations to retain N-specific antigenicity at elevated temperatures and that some ECs (PV53δ and PV57δ) were as stable as the current IPV.

### Conclusion.

Through thermal selection, we have shown that the PV-1 EC can be stabilized by mutations, e.g., in the VP1 pocket and at the 5-fold vertex. Selected variants were infectious and had thermally and antigenically stabilized ECs (stabilized PV-2 and PV-3 VLPs are in development). These capsid mutations can now be introduced into recombinant expression systems to synthesize antigenically stable potential PV VLP vaccines for a polio-free world.

## MATERIALS AND METHODS

### Virus and cells.

HeLa cells and mouse L cells were obtained from the National Institute of Biological Standards and Control, United Kingdom. The cDNA of wt PV-1 (strain Mahoney) used in this study was sourced from Bert Semler, University of California, and was cloned downstream of a T7 RNA promoter to allow *in vitro* RNA synthesis. A hammerhead ribozyme was included at the 5′ end, resulting in production of an authentic infectious PV-1 RNA (56). To initiate infection, L cells were transfected with PV-1 RNA, and the resulting viruses were propagated by standard methods (57, 58). Virus infectivity was determined by plaque assays, using HeLa monolayer cells (57). Virus titers were expressed as PFU per milliliter.

### Genome-free capsid preparation.

Virus was propagated in cysteine/methionine-deficient medium and radiolabeled with [^35^S]Cys/Met 2.75 h postinfection/transfection. At 3.25 h postinfection/transfection, the cells were treated with GuHCl at a final concentration of 2 mM to inhibit RNA replication and stimulate accumulation of genome-free ECs (28). The ECs were purified through 15 to 45% sucrose density gradients (28) and detected by scintillation counting of gradient fractions (59).

### Virus purification.

Virus-infected HeLa cells or RNA-transfected L cells were freeze-thawed and clarified by differential centrifugation. The supernatant was collected, and virus was pelleted though a 30% (wt/vol) sucrose cushion at 300,000 × *g* (using a Beckman SW55 Ti rotor) for 3.5 h at 4°C. The virus pellet was resuspended in phosphate-buffered saline (PBS) and clarified by differential centrifugation. The supernatant was purified through a 15 to 45% (wt/vol) sucrose density gradient by ultracentrifugation at 300,000 × *g* (using a Beckman SW55 Ti rotor) for 50 min at 4°C (28). The peak fractions corresponding to virions and ECs were collected. The respective peak fractions were pooled and diluted, and the particles were pelleted by ultracentrifugation (using a Beckman SW55 Ti rotor) at 300,000 × *g* for 2 h at 4°C. The pellets were resuspended in 300 μl PBS and reclarified by centrifugation. The supernatants were repurified through a second 15 to 45% sucrose gradient at 300,000 × *g* for 50 min at 4°C, as described above.

### Thermal selection.

Virus samples were heated for 30 min at temperatures resulting in 99.99% loss in titer. The surviving virus was propagated at 37°C in HeLa cells by standard methods, and the infectivity titers of pre- and postheating samples were determined by plaque assays (57). Thermal selection was carried out sequentially until the pre- and postheating titers were approximately equal. Heat inactivation assays were repeated, followed by rounds of thermal selection at increasing temperatures of 51°C, 53°C, and 57°C until there was a severe fitness cost.

### Assessing genetic stability.

Mutants were serially passaged five times without selection pressure. Serially passaged samples were then heated at the respective selection temperatures (51°C, 53°C, and 57°C). Preheating and postheating virus samples were assayed for infectivity.

### Thermal-stability assays. (i) Thermal inactivation assay.

Virus samples were incubated at a range of temperatures for 30 min within a thermal cycler and immediately cooled to 4°C for 5 min. The heat-treated samples were titrated by plaque assays (57).

### (ii) PaSTRy.

The thermal stability of the virus particles was further characterized by a thermofluorometric dual dye-binding assay using the nucleic acid dye SYTO9 and the protein-binding dye SYPRO orange (both from Invitrogen). Reaction mixtures of 50 μl containing 1.0-μg double-purified and desalted virus fractions, 5 μM SYTO9, 150× SYPRO orange, and PaSTRy buffer (2 mM HEPES, 200 mM NaCl, pH 8) were mixed and ramped from 25 to 95°C, with fluorescence reads taken at 1°C intervals every 30 s within the Stratagene MX3005p quantitative-PCR (qPCR) system (36).

### Antigenic stability. (i) Immunoprecipitation assay.

Radiolabeled virions and ECs were purified by sucrose density gradients as previously described (28) and incubated at a range of temperatures for 30 min. Particles were immunoprecipitated as described by Vrijsen et al. (60) using monoclonal PV-1 antibodies: MAb 234, which binds to pentameric intervals at the 3-fold axis of the N/D form of the capsid, or MAb 1588, which binds to nonnative (H/C) capsids and intermediates (2, 14, 41). The immunoprecipitated particles were counted by scintillation.

### (ii) SDS-PAGE.

Immunoprecipitated samples were boiled in SDS-loading loading buffer (100 mM Tris-Cl, pH 6.8, 4% [wt/vol] SDS, 0.2% [wt/vol] bromophenol blue). Proteins were separated through a 12% polyacrylamide gel by electrophoresis (61).

### (iii) Autoradiography.

Proteins separated by SDS-PAGE were fixed in a solution of 25% (vol/vol) isopropanol, 10% (vol/vol) acetic acid, and 65% (vol/vol) double-distilled H_2_O (ddH_2_O). The radioisotope signal was amplified using a fluorometric solution (GE Healthcare; NAMP100), and gels were vacuum dried onto filter paper. The dried gels were exposed to X-ray film at −80°C prior to developing (62).

### Western blotting.

Cell-cultured viruses and intermediates were harvested as previously described. Virus proteins were dissociated and separated by SDS-PAGE using standard protocols (61) and immunoblotted (63) with a rabbit polyclonal antibody (SH-16) to identify P1 (structural) proteins or a mouse anti-VP1 8650 MAb (Millipore). A rabbit polyclonal antibody (3CD) was used to identify P3 (nonstructural) proteins.

### Transmission electron microscopy.

Purified virions and ECs were dialyzed from sucrose fractions into dialysis buffer (10 mM HEPES, 100 mM NaCl, 1 mM EDTA, 1 mM dithiothreitol [DTT], pH 6.4) and visualized by negative-staining transmission electron microscopy according to standard protocols (64).

### Virus RNA extraction and sequencing.

Native virion particles were immunoprecipitated, and total RNA was extracted with guanidinium thiocyanate-phenol-chloroform (65). The structural (P1) region was reverse transcribed using a region-specific reverse primer (CTTGGCCACTCAGGATGATT). The reverse-transcribed cDNA was amplified by PCR using copy-proof Phusion DNA polymerase, a 5′ untranslated region (UTR) upstream primer (TTAAAACAGCTCTGGGGTTGTAC), and a P1-specific downstream primer (CTTGGCCACTCAGGATGATT), following standard protocols. The PCR amplicons were gel purified and sequenced. A-tailing (addition of 3′ adenine residues) of PCR amplicons was carried out, followed by cloning into the high-copy-number pGEM-T Easy vector plasmid, and individual colonies were sequenced for confirmation (66).

### Mutagenized infectious clones.

Identified capsid mutations were introduced into the previously described wt PV-1 infectious clone by SDM (67). Escherichia coli DH5α cells were transformed with mutagenized plasmid constructs and selected for antibiotic resistance according to standard protocols (68). Colonies were randomly selected and sequenced to confirm the presence of introduced mutations. The plasmids were used as templates for RNA synthesis, and RNA was transfected into the cells as described above.

### Statistical analysis.

Where appropriate, data were analyzed by one- and two-way analysis of variance (ANOVA) for multitest analysis of multiple groups, while Student *t* tests were applied to comparison of single-test analysis of 2 or more groups. GraphPad Prism version 7.01 for Windows was used (GraphPad Software, La Jolla CA).
